# Dental Caries among Adult Population of a Municipality: A Descriptive Cross-sectional Study

**DOI:** 10.31729/jnma.7807

**Published:** 2022-10-31

**Authors:** Anju Khapung, Shrijana Shrestha

**Affiliations:** 1 Department of Community and Public Health Dentistry, Nepal Medical College and Teaching Hospital, Jorpati, Kathmandu, Nepal; 2 Department of Oral Medicine and Radiology, Kantipur Dental College Teaching Hospital & Research Center, Basundhara, Kathmandu, Nepal

**Keywords:** *cross-sectional study*, *dental decay*, *prevalence*

## Abstract

**Introduction:**

Oral disease as a public health problem poses a serious burden globally. The most common oral disease affecting adults is dental caries followed by periodontal disease leading to tooth loss. Early detection of dental caries can help reduce the severity and prevent further complications. This study aimed to find out the prevalence of dental caries among adult population of a municipality.

**Methods:**

This descriptive cross-sectional study was conducted among adults attending five different dental camps in a municipality from 1 April 2022 to 2 June 2022. Ethical approval was obtained from Institutional Review Committee (Reference number: 060-078/079). Convenience sampling method was used. The prevalence of dental caries was determined by dentition status adopted from basic oral health surveys recommended by World Health Organization. Point estimate and 95% Confidence Interval were calculated.

**Results:**

Among 239 adults, 138 (57.74%) (51.48-64, 95% Confidence Interval) had dental caries.

**Conclusions:**

The prevalence of dental caries among adults in the municipality was lower than in similar studies done in similar settings.

## INTRODUCTION

Globally, oral diseases have been categorized as the fourth most expensive disease to treat. Oral conditions affected 3.9 billion people, and untreated caries in permanent teeth was the most prevalent condition evaluated for the entire Global Burden of Disease 2010.^1^ Despite being highly preventable, a substantial proportion of the Nepalese population, especially poor and marginalized individuals, experience oral health problems.^2,3^ One-fourth of Nepalese adults have been found to have self-reported dental caries.^4^ A past study done in Nepal showed that majority of the adults had tooth loss due to dental caries.^5^

As dental caries is one of the major oral health problems seen among the adults, early detection can help reduce the severity and prevent further complications. Data obtained could also help in planning preventive and curative dental programs for the target population. Any relevant literature among adult population in the municipality could not be assessed.

This study aimed to find out the prevalence of dental caries prevalence among adult population of a municipality.

## METHODS

This descriptive cross-sectional study was conducted among the adults residing in Gokarneshwar municipality who attended five different dental camps conducted by the Department of Community and Public Health Dentistry, Nepal Medical College and Teaching Hospital between 1 April 2022 to 2 June 2022. Ethical approval was obtained from the Institutional Review Committee of the same institute (Reference number: 060-078/079) and informed consent was taken from all the study participants prior to the study.

Study participants aged 18 years and above and who were willing to participate were included in the study and the participants who did not provide consent were excluded from the study. Convenience sampling method was used. The sample size was calculated by using the following formula:


$$\begin{array}{ll} \text{n} & ={\text{Z}}^{2}\times \frac{\text{p}\times \text{q}}{{\text{e}}^{\text{2}}}\\ & =1.96^{2}\times \frac{0.50\times 0.50}{0.07^{2}}\\ & =196 \end{array}$$

Where,

n= minimum required sample size
Z= 1.96 at 95% Confidence Interval (CI)
p= prevalence taken as 50% for maximum sample size calculation
q= 1-p
e= margin of error, 7%

In the calculated sample size, 10% was added to address the non-response rate after which the required sample size was 216. However, a total of 239 adults were included in the study.

Data related to socio-demographic details were obtained. The study participants were categorized into three age groups: 18-35 years (young adults), 36-55 years (middle-aged adults) and 56 years and above (older adults).^6^ Clinical examination was done to assess the dentition status and treatment needs as adopted from basic oral health surveys recommended by World Health Organization (WHO) using a mouth mirror and explorer.^7^ For the assessment of dental caries, the decayed components included code 1 (decayed teeth) and 2 (filled teeth with decay) from the dentition status code and criteria.

Teeth were considered to be decayed when there was obvious cavitation, discoloration typical of undermined enamel, explorer tip penetrated deep into soft yielding material, and explorer tip in a pit or fissure resisted removal after moderate to firm pressure on insertion.^8^ Treatment needs for dental caries included scores 1 (one surface filling), 2 (two or more surface filling), 5 (pulp care and restoration), and 6 (extraction). Autoclaved instruments were used for clinical examination. The chemical method of disinfection was done by using Cidex (glutaraldehyde solution) in the study site in needed situations. The oral conditions that required urgent treatment were referred to nearby dental care centres.

Data were entered in Microsoft Excel and exported to IBM SPSS Statistics 20.0 for analysis. Point estimate and 95% CI were calculated.

## RESULTS

Among 239 adults, 138 (57.74%) (51.48-64, 95% CI) had dental caries. Decayed teeth was seen in 135 (97.82%) adults (Table 1).

**Table 1:** Code and criteria from dentition status of teeth with dental caries (n= 138).

Code and criteria	n (%)
Code 1 (decayed teeth)	135 (97.82)
Code 2 (filled teeth with decay)	8 (5.79)

One surface filling was done in 106 (76.81%) followed by extraction in 52 (37.68%) participants (Table 2).

**Table 2:** Treatment needs among the adults with dental caries (n= 138).

Treatment needs	n (%)
One surface filling	106 (76.81)
Two or more surface fillings	22 (15.94)
Pulp care and restorations	13 (9.42)
Extraction	52 (37.68)

The age ranged from 18 to 83 years with a mean age of 35.75±15.76, 38.60±17.21 and 34.51±15 years among all adults, males, and females respectively. Of the total 138 participants with dental caries, 82 (59.42%) belonged to the 18-35 years age group and 96 (69.57%) were females (Table 3).

**Table 3:** Demographic characteristics of adults with dental caries (n= 138).

Variables	n (%)
**Age (years)**	
18-35	82 (59.42)
36-55	41 (29.71)
>56	15 (10.87)
**Sex**	
Male	42 (30.43)
Female	96 (69.57)

## DISCUSSION

Dental caries has now become a public health problem regardless of age. Though dental caries has been found to affect young children, it also tracks across adolescence, adulthood, and into later life persisting as a chronic and progressive condition.^9^ Majority of the epidemiological studies done in Nepal have been conducted mainly on children rather than adults. This descriptive cross-sectional study had been conducted in the community among adults encompassing different areas of Gokarneshwar municipality and including varied age groups to obtain data regarding dental caries prevalence.

In this study, the prevalence of dental caries among adults was found to be 57.74%. In another study done in Nepal, the prevalence was found to be 57.50%, a finding similar to our present study.^10^ However this finding is lower than a study done in Saptari, Nepal, where 83% of adults had dental caries.^11^ This difference could be due to the larger sample size and different sampling method adopted in that study. In a study done in India, the prevalence of dental caries was higher.^12^ This difference in prevalence could be due to longer study duration and data being collected in institutional settings where most patients with dental problems visit. The present study was conducted among adults from five dental camps only and this could have been the reason of the lower prevalence.

In the current study, the majority of the adults with dental caries belonged to the 18-35 years age group and they had a dental caries prevalence of 59.42%. In a study done in Egypt,^13^ the prevalence of dental caries among 18-34 years adults was also 58.38% and the dental caries prevalence decreased with an increase in age, the findings almost similar to our study. Decreasing incidence of dental caries with increasing age was also found in various other studies.^12,14^ This finding provide us an area for further research to find out factors attributable to this difference.

In the present study, a greater number of females had dental caries than males. Studies done in different countries have also shown this gender difference in caries prevalence, female having a higher prevalence than males.^15-17^ This could be due to risk factors among women like different salivary composition and flow rate, genetic variations, dietary habits, hormonal effects, psychosocial and economic factors and earlier teeth eruption pattern in females leading to longer exposure of teeth to oral environment.^18-20^ Extraction was the treatment need among 37.68% of the adults in our study. A similar finding has been observed in a few other studies.^10,21^

The present study is one of the few studies conducted to find out the prevalence of dental caries among Nepalese adults. However, the use of the convenience sampling method in this study could limit generalizability for all adults in Nepal. Further studies with a larger sample size and probability sampling methods are recommended.

## CONCLUSIONS

The prevalence of dental caries among adults was lower than in published studies done in similar settings. However, this prevalence suggests the need for planning preventive and promotive oral health programs for the adult population.
